# Using a Bayesian latent variable approach to detect pleiotropy in the Genetic Analysis Workshop 18 data

**DOI:** 10.1186/1753-6561-8-S1-S77

**Published:** 2014-06-17

**Authors:** Lizhen Xu, Radu V Craiu, Andriy Derkach, Andrew D Paterson, Lei Sun

**Affiliations:** 1 Department of Statistical Sciences, University of Toronto, Toronto, Ontario M5S 3G3, Canada; 2 Program in Genetics and Genome Biology, The Hospital for Sick Children, Toronto M5G 1X8, Canada; 3 Division of Biostatistics, Dalla Lana School of Public Health, University of Toronto, Ontario M5S 3G3, Canada

## Abstract

Pleiotropy, which occurs when a single genetic factor influences multiple phenotypes, is present in many genetic studies of complex human traits. Longitudinal family data, such as the Genetic Analysis Workshop 18 data, combine the features of longitudinal studies in individuals and cross-sectional studies in families, thus providing richer information about the genetic and environmental factors associated with the trait of interest. We recently proposed a Bayesian latent variable methodology for the study of pleiotropy, in the presence of longitudinal and family correlation. The purpose of this work is to evaluate the Bayesian latent variable method in a real data setting using the Genetic Analysis Workshop 18 blood pressure phenotypes and sequenced genotype data. To detect single-nucleotide polymorphisms with pleiotropic effect on both diastolic and systolic blood pressure, we focused on a set of 6 single-nucleotide polymorphisms from chromosome 3 that was reported in the literature to be significantly associated with either diastolic blood pressure or the binary hypertension trait. Our analysis suggests that both diastolic blood pressure and systolic blood pressure are associated with the latent hypertension severity variable, but the analysis did not find any of the 6 single-nucleotide polymorphisms to have statistically significant pleiotropic effect on both diastolic blood pressure and systolic blood pressure.

## Background

Abundant pleiotropy has been reported for complex human traits [1]. However, few current genetic studies formally investigate pleiotropy, because of the analytical difficulties in modeling the inherent data complexity. Challenging aspects include the different types of phenotype of interest (continuous, discrete, or both) and various correlations present in the data (between the phenotypes of interest, between individuals if family data are collected, and between measurements over time if longitudinal data are collected).

There have been some recent developments in methods proposed for joint analysis of multiple phenotypes. For example, Weller et al [2] applied principal component analysis to multiple traits to obtain independent canonical variables and then conducted univariate quantitative trait locus (QTL) analyses. Lange and Whittaker [3] developed a QTL-mapping method based on generalized estimating equations. Xu et al [4] extended the standard linear combination test to incorporate data-driven weighting factors. Nicolae et al [5] studied the correlation between 2 quantitative traits stratified by genotype. Borecki et al [6] investigated the percentage of trait correlation explained by a marker of interest. O'Reilly et al [7] reversed the role of phenotype and genotype so that the genotype is treated as the response variable. Li et al [23] modified the approach of O'Reilly et al [7] and proposed a likelihood ratio test that compares a full model with 2 phenotypes of interest with a nested model. However, these methods were proposed primarily for studies of quantitative traits with cross-sectional data in unrelated individuals.

Longitudinal family data, such as Genetic Analysis Workshop 18 (GAW18), combine the features of longitudinal studies in independent individuals and studies using families. Therefore, they provide more information about the genetic and environmental factors associated with the trait of interest than cross-sectional studies [8]. However, joint modeling of multiple phenotypes using longitudinal family data involves nontrivial analytical challenges because of the complex phenotypic, familial, and serial correlations.

We recently proposed to use the latent variable (LV) methodology for study of pleiotropy, in the presence of longitudinal and family correlation [9]. The LV methodology has been widely used in many scientific fields, including economics, psychology, and social sciences, and it is becoming increasingly attractive for genetic studies. For example, Ohara et al [10] proposed a LV approach for the analysis of multivariate quantitative trait loci; Tayo et al [11] applied a factor analysis (a subtype of the LV model [LVM]) to find latent common genetic components of obesity traits; and Nock et al [12] used factor analysis for a metabolic syndrome study. Initial applications of LVM focused on reducing the number of manifest variables to a smaller number of latent outcomes. Sammel and Ryan [13,14] extended the LVM to allow covariates to have effects on both the manifest and LVs. Roy and Lin [15] discussed a LV approach for longitudinal data with continuous outcomes. Xu et al [9] extended the work of Roy and Lin [15] to accommodate both binary and continuous phenotypes and family data, and proposed a Bayesian approach for parameter estimation. The focus of this work is to evaluate the Bayesian LV method of Xu et al [9] in a real data setting using the GAW18 blood pressure phenotype data and sequenced genotype data.

## Methods

### The latent variable model

Here we briefly review the LV methodology proposed by Xu et al [9]. The formulation of the LVM relies on postulating the effect of a random variable that is not observed by the researchers but is assumed to play an important role in various observed variables (also known as the manifest variables), and thus induces correlations among them [16]. In the context of pleiotropy studies, the manifest variables are the multiple observed phenotypes, which inform the LV that represents the underlying conceptual disease status or severity. The proposed LVM consists of 2 parts. The first part models the relationship between the manifest variables (*Y *s) and the LV (*U*) to characterize the within-subject correlation among different outcomes. The second part uses a linear mixed-effect model to investigate the effect of a genetic marker and other covariates (*X *s) on the LV (*U*), accounting for the longitudinal and familial correlations.

Formally, let $Y_{cit}={\left(y_{cit1},\ldots ,y_{citJ}\right)}^{\prime }$ be the *J * × 1 vector of responses (eg, phenotypes) measured at the *t^th ^*time on the *i^th ^*individual from the *c^th ^*family (or cluster) for c=1,2,…,C, $i=1,2,\ldots ,N_{c}$, $t=1,2,\ldots ,T_{ci}$, where *C *denotes the total number of families, *N_c _*is the number of individuals within the *c^th ^*family, *T_ci _*is the total number of repeated measurements for the individual {*c, i*} and *J *is the total number of responses. Among the *J *responses, $Y_{cit}^{c}={\left(y_{cit1}^{c},\cdots \;,y_{citJ_{1}}^{c}\right)}^{\prime }$ are the continuous responses and $Y_{cit}^{b}={\left(y_{citJ_{1}+1}^{b},\cdots \;,y_{citJ}^{b}\right)}^{\prime }$ are the binary responses. Let *U_cit _*be the LV representing the conceptual disease severity, which summarizes the partial information brought by each of the *J *phenotypes.

The first part of the LVM models the relationship between the phenotypes (*Y*s) and the LV (*U*) to characterize the within-subject correlation among the different outcomes. A continuous phenotype is linked to the latent trait *U_cit _*via a linear mixed model

(2.1) $$y_{citj}^{c}=\beta_{0j}+W_{cit}^{T}\beta_{j}+\lambda_{j}U_{cit}+b_{cij}+e_{citj}$$

where $e_{citj}\overset{iid}{\sim }N\left(0,\sigma_{j}^{2}\right)$, *W_cit _*is a *p*_1_-dimensional vector of direct effect covariates, $\beta_{0j}$ is the mean effect of phenotype *j*, and $\lambda_{j}$ is the factor loading that represents the effect of the LV on the phenotype. The random component *b_cij _*captures the family-specific within-subject correlations over time. We assume $b_{cij}\overset{iid}{\sim }N\left(0,\tau_{j}^{2}\right)$, and *e_citj _*and *b_cij _*are mutually independent. If a phenotype is binary, a generalized linear mixed model,

(2.2) $$\eta_{citj}=\beta_{0j}+W_{cit}^{T}\beta_{j}+\lambda_{j}U_{cit}+e_{citj}$$

is assumed with a probit link,

(2.3) $$E\left[y_{citj}^{b}|U_{cit},b_{cij}\right]=p\left(y_{citj}^{b}=1|U_{cit},b_{cij}\right)=\text{Φ}\left(\eta_{citj}\right)$$

We choose a probit link instead of a logit link to gain computational efficiency.

The second part of the LVM models the influence of indirect covariates on the LV via the linear mixed model,

(2.4) $$U_{cit}=X_{cit}^{T}\alpha +a_{c}+d_{ci}+\varepsilon_{cit}$$

where $\epsilon_{cit}\overset{iid}{\sim }N\left(0,1\right)$, *a_c _*and *d_ci _*are the random effects to model correlations of the LV *U_cit _*among subjects within a family and the dependency between repeated measures of *U_cit_*, with $a_{c}\sim N\left(0,\sigma_{a}^{2}\right)$, and **$d_{ci}\sim N\left(0,\sigma_{d}^{2}\right)$**. *X_cit _*is a *p*_2_-dimensional vector of covariates that can include both environmental and genetic factors, and *α *is the corresponding vector of regression parameters characterizing the association between the LV (*U*) and *X*. This set of covariates influences the phenotypes through its impact on the LV (*U*) and is often of the primary interest. Particularly, if a single-nucleotide polymorphism (SNP) included in *X *is found to be statistically associated with *U*, the SNP is deemed to be pleiotropic. For the purpose of model identification, we assume that the 2 sets of covariates *X *and *W *are disjoint.

### Parameter estimation via Bayesian method

Xu et al [9] considered a Bayesian estimation for the LVM parameters to allow a principled approach to incorporate prior information, which can be substantial in many practical genetic studies, and to produce finite sample likelihood-based inference. The data in the proposed LVM contain the observed continuous and binary outcomes *Y*, the covariates *W *with direct effect on *Y*, and the covariates *X *with indirect effect on *Y *via *U*. The parameter of interest is $\text{Θ}={\left(\beta_{0},\beta ,\alpha ,\lambda ,\tau^{2},\sigma^{2},\sigma_{a}^{2},\sigma_{d}^{2}\right)}^{\prime }$ where $\beta_{0}={\left(\beta_{01},\cdots \;,\beta_{0J}\right)}^{\prime }$, $\beta ={\left(\beta_{1}^{\prime },\ldots ,\beta_{J}^{\prime }\right)}^{\prime }$, $\beta_{j}={\left(\beta_{j1},\ldots ,\beta_{jp_{1}}\right)}^{\prime }$$\alpha ={\left(\alpha_{1},\ldots ,\alpha_{p2}\right)}^{\prime }$, $\lambda ={\left(\lambda_{1},\ldots ,\lambda_{J}\right)}^{\prime }$, $\tau^{2}={\left(\tau_{1}^{2},\cdots \;,\tau_{J}^{2}\right)}^{\prime }$ and $\sigma^{2}={\left(\sigma_{1}^{2},\cdots \;,\sigma_{J_{1}}^{2}\right)}^{\prime }$. The posterior distribution for the model parameters is:p(Θ|Y,W,X)∝P(Θ)p(Y|Θ,W,X) Markov chain Monte Carlo (MCMC) algorithms are used to draw posterior samples for statistical inference. In addition, the data augmentation principle of Tanner and Wong [17] and the parameter expansion and hierarchical centering techniques of Gelfand [18], Liu and Wu [19], and Meng and van Dyk [20] are applied to speed up the MCMC's mixing. Uninformative conjugate priors are given to the parameters in the expanded model. Bayes factors (BFs) calculated using the path sampling method are used to test the significance of factor loadings and the fixed effects. The support for the alternative hypothesis of pleiotropy is detected if log BF > 0.5 [9].

### Data analysis

The phenotypes of interest are diastolic blood pressure (DBP) and systolic blood pressure (SBP). We considered 464 individuals from the 16 families (Type 2 Diabetes Genetic Exploration by Next-generation sequencing in Ethnic Samples [T2D-GENES] Project 2) that were sequenced. Among the 464 individuals, 396 individuals have at least 1 blood pressure measure (90 have only 1, 78 have 2, 131 have 3, and 97 have 4 measurements). The length of time between 2 consecutive measurements ranges from 3 to 9 years, and the number of family members varies from 11 to 36. For the phenotypes of interest, we added a value of 15 to SBP and a value of 10 to DBP for those individuals who took antihypertensive medication [21]. Among the available covariates--age, sex, antihypertensive medications, and tobacco smoking status--we included age and sex in our analysis.

To detect SNPs with pleiotropy effect, we focused on a set of 6 SNPs from chromosome 3 (rs9816772, rs9852991, rs6768438, rs9815354, rs7640747 and rs743395) that had been reported to be significantly associated with either DBP or the binary hypertension trait, based on a large-scale genome-wide association study of blood pressure and hypertension under an additive genetic model assumption [22]. Table 1 summarizes the results that were reported in Levy et al [22]. It shows that 4 SNPs (rs9816772, rs9852991, rs6768438, and rs9815354) were found to be significantly associated with DBP but not with SBP nor with the binary hypertension trait. Note that the 4 SNPs are near the gene *ULK4*, close to each other, and likely to be in strong linkage disequilibrium (LD). Therefore their statistical association results are expected to be similar. The other 2 SNPs (rs7640747 and rs743395) were found to be significantly associated with hypertension, but only suggestively with SBP or DBP. These 2 SNPs are also close to each other in high LD and near ITGA9.

**Table 1:** Genome-wide association result for the 6 SNPs reported by Levy et al [22]

			*p *Values		
			
SNP	Gene	MAF	DBP	SBP	Hypertension
rs9816772	*ULK4*	0.16	9.7 × 10^−7^	0.5	0.85
rs9852991	*ULK4*	0.16	9.7 × 10^−7^	0.59	0.85
rs6768438	*ULK4*	0.16	9.7 × 10^−7^	0.59	0.84
rs9815354	*ULK4*	0.17	7.8 × 10^−7^	0.69	0.83
rs7640747	*ITGA9*	0.38	9.5 × 10^−4^	5.9 × 10^−4^	4.8 × 10^−7^
rs743395	*ITGA9*	0.38	4.4 × 10^−4^	8.0 × 10^−4^	7.5 × 10^−7^

In the GAW18 data analyzed, the minor allele frequency (MAF) for these SNPs are 0.15, 0.13, 0.18, 0.13, 0.30, 0.29, respectively.

We then applied the Bayesian LVM method to analyze 1 SNP at a time, assuming an additive genetic model. For each SNP of interest, the phenotypes *Y*s are SBP and DBP, the covariates include the genotype of the SNP, age, and sex, and the continuous LV *U *represents the conceptual hypertension severity level. We began our analysis by considering all possible models that include the 3 covariates, where each covariate has either direct effects on the phenotypes *Y*s or indirect effect through *U*. Table 2 gives the deviance information criteria statistics of these models and model 5 (covariate Sex is included in Equation (2.1) with direct effect on the phenotypes and Age and SNP in Equation (2.4) with indirect effect on the phenotypes via the LV) has smallest deviance information criteria (DIC) value. Thus, we used model 5 to fit the data. We then applied the BF method to further determine whether the effects of these chosen covariates are statistical significant. The folded-t prior with 3 degrees of freedom is used for $\lambda_{j}$, $N_{p1}\left(0,I_{p1}\right)$ is used for $\beta_{j}$, and $N_{p2}\left(0,{\text{I}}_{p2}\right)$ is used for *α*. For comparison, we also report the highest posterior density interval (HpdI) for testing the factor loadings and the fixed effects.

**Table 2:** Goodness-of-fit statistics for alternative latent variable models applied to rs9816772

Model	Covariates		DIC
		
	With phenotypes *Y *s in Equation (2.1)	With latent variable *U *in Equation (2.4)	
1	Age+sex+SNP	N/A	15388.7
2	Age+sex	SNP	15729.3
3	Age+SNP	Sex	15746.5
4	Sex+SNP	Age	15820.6
5	Sex	Age+SNP	15226.5
6	SNP	Age+sex	15247.9
7	Age	Sex+SNP	15744.0
8	N/A	Age+sex+SNP	15948.3

*DIC*, deviance information criteria.

## Results

Based on the results shown in Table 3, both SBP and DBP are associated with the latent hypertension severity variable with the factor loading for SBP being 13.15 and for DBP being 7.60, which are all significantly larger than zero. Furthermore, the sizes of the 2 factor loadings suggest that the strength of the relationship between SBP and the LV is about 1.7 times greater than that between DBP and the LV. Sex shows no significant association with SBP, with the logarithm of Bayes factor (logBF) being negative, and the 95% HpdI covering zero. However, it has significant negative effect on DBP, indicating that females have lower DBP than males. Age appears to have a significant positive effect on the underlying hypertension severity variable with logBF equaling 126.53. There is no evidence that rs9816772 is significantly associated with the LV, with the logBF being less than zero and the 95% HpdI for *α*_1_, the coefficient for the SNP, covering zero. Results for other SNPs are characteristically similar to what's reported here for rs9816772. Therefore, our analysis did not detect a pleiotropy effect on both DBP and SBP for any of the 6 SNPs that were previously reported to be associated with DBP or the binary hypertension trait.

**Table 3:** Results of the Bayesian LV method applied to rs9816772, previously identified as associated with DBP

	Parameter	Estimate	logBF	95% HpdI
SBP	$\lambda_{1}$	13.15	255.3	(12.19, 14.11)
DBP	$\lambda_{2}$	7.60	139.6	(7.01, 8.14)
Sex for SBP	$\beta_{11}$	−0.66	−0.074	(−2.12, 0.81)
Sex for DBP	$\beta_{21}$	−1.79	2.017	(−2.92, -0.65)
rs9816772	$\alpha_{1}$	−0.045	−0.653	(−0.208, 0.124)
Age	$\alpha_{2}$	0.043	126.53	(0.036, 0.049)

$\lambda_{j}$ is the factor loading for the association between phenotype *Y_j _*(*j *= 1, 2: SBP and DBP) and the conceptual latent variable *U*, $\beta_{jk}$ evaluates the association between phenotype *Y_j _*and covariate *W_k_*( *k *= 1: sex), $\alpha_{k}$ captures the association between the latent variable *U *and covariate *X_k _*(*k *= 1, 2: SNP and age).

## Discussion and conclusions

In this paper, we investigated the Bayesian LV methodology recently proposed by our group to joint model multiple phenotypes, in the presence of serial and familial correlations [9]. The proposed method provides a conceptually easy but effective way to jointly study multiple correlated phenotypes, which could be a mixture of continuous and binary outcomes with serial and familial correlations. These multiple outcomes are assumed to be related to a LV, which can be interpreted as the latent disease severity. As a by-product of our MCMC algorithm, the method provides subject-specific estimate of the LV, which can be used for further analysis. The 2-level modeling scheme allows us to estimate and test the global effects of the covariates on the multiple outcomes, which are more efficient than the traditionally used multiple tests [15].

We applied this method to the GAW18 data, jointly analysing SBP and DBP. A LV *U *is introduced to characterize the within-subject correlation between the 2 phenotypes and can be interpreted as the underlying hypertension severity variable. Pleiotropy is detected if a genetic marker is found to have a significant effect on *U*. To detect SNPs with pleiotropic effect on SBP and DBP, we investigated a set of 6 SNPs from chromosome 3 that were previously shown to be significantly associated with either DBP or the hypertension trait. However, we did not see statistically significance evidence for pleiotropy effect for any of the 6 SNPs.

The incorporation of family-specific random effect *a_c _*in equation (2.4) allows us to model the familial correlation of the underlying disease trait. However, this formulation does not take into account the different degrees of genetic relationship among the individuals within a family. Thus, alternative models incorporating the kinship matrix will be considered in the future. Our future work also includes developing alternative models that adjust for the unequally spaced time measurements, and more efficient algorithms so that a genome-wide search for pleiotropic SNPs can be performed. (The current computation time is about 1.7 minutes per SNP.) Evaluating the performance of the Bayesian LVM method using the simulated data, and extension of the method to joint analysis of multiple rare variants are also of interest.

## Competing interests

The authors declare that they have no competing interests.

## Authors' contributions

Study design: LX, RVC, ADP, and LS. Analysis: LX and AD. Manuscript drafting: LX and LS. All authors read and approved the final manuscript.
